# *Enterococcus faecalis* and pathogenic streptococci inactivate daptomycin by releasing phospholipids

**DOI:** 10.1099/mic.0.000529

**Published:** 2017-09-25

**Authors:** Elizabeth V. K. Ledger, Vera Pader, Andrew M. Edwards

**Affiliations:** MRC Centre for Molecular Bacteriology and Infection, Imperial College London, Armstrong Rd, London, SW7 2AZ, UK

**Keywords:** *Staphylococcus*, *enterococcus*, *streptococcus*, daptomycin, phospholipid

## Abstract

Daptomycin is a lipopeptide antibiotic with activity against Gram-positive bacteria. We showed previously that *Staphylococcus aureus* can survive daptomycin exposure by releasing membrane phospholipids that inactivate the antibiotic. To determine whether other pathogens possess this defence mechanism, phospholipid release and daptomycin activity were measured after incubation of *Staphylococcus epidermidis*, group A or B streptococci, *Streptococcus gordonii* or *Enterococcus faecalis* with the antibiotic. All bacteria released phospholipids in response to daptomycin, which resulted in at least partial inactivation of the antibiotic. However, *E. faecalis* showed the highest levels of lipid release and daptomycin inactivation. As shown previously for *S. aureus*, phospholipid release by *E. faecalis* was inhibited by the lipid biosynthesis inhibitor platensimycin. In conclusion, several pathogenic Gram-positive bacteria, including *E. faecalis,* inactivate daptomycin by releasing phospholipids, which may contribute to the failure of daptomycin to resolve infections caused by these pathogens.

Daptomycin is a lipopeptide antibiotic that is used as a last resort in the treatment of infections caused by methicillin-resistant *Staphylococcus aureus* (MRSA), vancomycin-intermediate *S. aureus* (VISA) and vancomycin-resistant enterococci (VRE) [1–3]. The use of daptomycin is becoming more common, with prescriptions increasing by 72 % between 2012 and 2015 in the UK [4]. Daptomycin is the only lipopeptide antibiotic that is used clinically and it functions in a similar manner to antimicrobial peptides [5]. The antibiotic inserts into the membrane of Gram-positive bacteria by targeting phosphatidylglycerol, where it forms oligomeric complexes [6–8]. The precise mechanism by which the antibiotic kills bacteria is unclear, but it involves depolarization of the bacterial membrane and inhibition of cell wall biosynthesis without causing lysis [8–13]. Although daptomycin resistance is rare, treatment failure occurs in up to 30 % of staphylococcal infections and 23 % of enterococcal infections [14, 15]. The failure rates are highest in invasive infections such as bacteraemia or osteomyelitis, with rates of 24 and 33 % respectively, resulting in poor patient prognoses [14]. Understanding the reasons for this treatment failure is crucial to improving the effectiveness of daptomycin treatment.

We recently discovered that *S. aureus* has a transient defence mechanism against daptomycin, which contributed to treatment failure in a murine model of invasive infection [16]. In response to the antibiotic, phospholipids were released from the cell membrane, which sequestered daptomycin and abrogated its bactericidal activity [16]. Phospholipid release occurred via an active process, which was blocked by the lipid biosynthesis inhibitor platensimycin [16, 17]. In addition to daptomycin, phospholipid release also provided protection against the antimicrobial peptides nisin and melittin, suggesting a general defence against membrane-targeting antimicrobials [16].

It is currently unknown whether other Gram-positive bacteria release phospholipids in response to daptomycin, although membrane vesicles have been observed on the surface of *Enterococcus faecalis* cells exposed to daptomycin [18]. In addition, there is growing evidence that other Gram-positive pathogens, including group A streptococci (GAS) and group B streptococci (GBS), release phospholipids from their surfaces in the form of extracellular vesicles [19, 20]. Production of these membrane vesicles is increased in the presence of antimicrobials and, at least for GAS, they are rich in phosphatidylglycerol, which was shown to be essential for daptomycin inactivation by *S. aureus* [16, 19, 21]. Therefore, we hypothesized that phospholipid release is a common strategy amongst Gram-positive pathogens to resist membrane-acting antimicrobials.

Given the increasing use of daptomycin to treat enterococcal infections, the primary aim of this work was to determine whether enterococci release membrane phospholipids that inactivate the antibiotic. We also examined pathogenic streptococci and *S. epidermidis*, as the rising tide of antibiotic resistance may necessitate the use of daptomycin to tackle these bacteria in the future.

We initially determined the daptomycin minimum inhibitory concentration (MIC) for a representative panel of Gram-positive pathogens: *S. aureus* SH1000 [22], *S. epidermidis* ATCC 12228 [23], GAS strain A40 [24]; GBS strains 515 [25] and COH1 [26]; *S. gordonii* strain Challis [27]; and *E. faecalis* strains JH2-2 [28] and OG1X [29]. All bacteria were grown in Müller–Hinton broth and either brain heart infusion broth (BHI) for the enterococci and streptococci or tryptic soy broth (TSB) for the staphylococci, each containing calcium (0.5 mM). The MIC was then determined by the broth microdilution approach [30]. The most susceptible species, with the lowest MIC values (MHB/BHI or TSB), were the pathogenic GAS strain A40 (0.125/0.125 µg ml^−1^), and GBS strains 515 (0.5/0.25 µg ml^−1^) and COH1 (0.5/0.5 µg ml^−1^), whilst *S. aureus* (1/1 µg ml^−1^), *S. epidermidis* (1/1 µg ml^−1^), *S. gordonii* Challis (2-4/4 µg ml^−1^), and *E. faecalis* strains OG1X (2/2 µg ml^−1^) and JH2-2 (4/2 µg ml^−1^) were the least susceptible.

To determine whether *E. faecalis* or streptococci respond to daptomycin by releasing membrane phospholipids, we exposed streptococci and enterococci (10^8^ c.f.u. ml^−1^) to various supra-MIC concentrations of the antibiotic (5–40 µg ml^−1^) in BHI (0.5 mM CaCl_2_) broth at 37 °C under static conditions with 5 % CO_2_ and measured bacterial survival, antibiotic activity and phospholipid release (Fig. 1c–h, k–p, s–x). Staphylococci were also exposed to daptomycin (5–40 µg ml^−1^), but in TSB containing 0.5 mM CaCl_2_ at 37 °C with shaking (180 r.p.m.) (Fig. 1a, b, i, j, q, r).

**Fig. 1.**
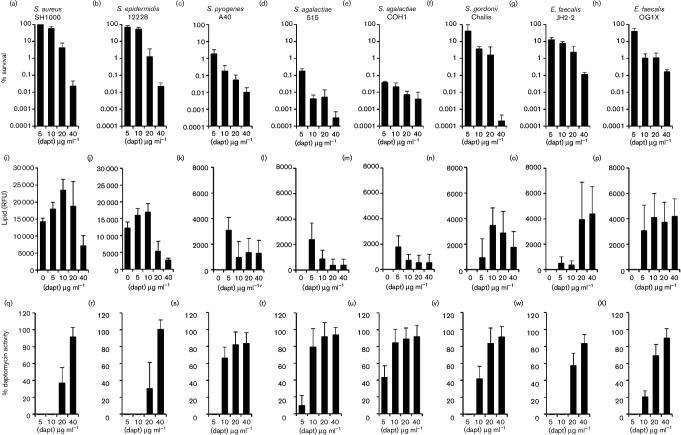
Streptococci and enterococci release phospholipids and inactivate daptomycin. (a–h) Percentage survival of bacteria after 8 h incubation in broth containing the indicated concentrations of daptomycin. (i–p) The concentration of phospholipid in culture supernatants of bacteria exposed to daptomycin, as determined by reactivity with a fluorescent dye (RFU, relative fluorescence units). Note the different *y*-axis scale for staphylococci vs other bacteria. (q–x) Relative percentage of daptomycin activity remaining in culture supernatants of bacteria exposed to daptomycin for 8 h. The activity of daptomycin incubated in culture medium only for 8 h was taken to be 100 %. For all data, the mean of four independent experiments is shown, and the error bars represent the sd of the mean.

For all strains, there was a dose-dependent decrease in survival after 8 h exposure to daptomycin, as assessed by c.f.u. counts (Fig. 1a–h). Broadly, the survival of strains exposed to supra-MIC concentrations of daptomycin correlated with the MIC values, with survival of the two enterococcal strains, the staphylococci and *S. gordonii*, being greater than the survival of the GAS or GBS strains at each of the concentrations of daptomycin examined (Fig. 1a–h).

Next, we explored whether streptococci and enterococci released phospholipids in response to daptomycin challenge, and how this related to the susceptibility of the strains to the antibiotic. Using the phospholipid-reactive fluorescent dye FM-4-64 (Life Technologies), we confirmed our previous observation that wild-type staphylococci released phospholipids in the absence of daptomycin, but this was significantly increased for bacteria exposed to daptomycin (Fig. 1i, j) [16]. By contrast, neither enterococci nor streptococci released phospholipids in the absence of daptomycin (Fig. 1k–p). Upon exposure to daptomycin, however, all of the streptococci and enterococci released phospholipids, albeit to differing levels. The quantity of phospholipid released was much greater for staphylococci than for the other species examined (Fig. 1i–p). However, for both staphylococci and streptococci, the quantity of phospholipid released was lowest when the daptomycin concentration was highest, suggesting that the antibiotic may have killed the bacteria before they could release the lipid (Fig. 1i–p). By contrast, the enterococci released high levels of phospholipid in the presence of the highest concentrations of daptomycin (Fig. 1o, p). Therefore, unlike survival, phospholipid release did not correlate with daptomycin MIC, and this may indicate different daptomycin concentration thresholds for the triggering of phospholipid release.

To determine whether phospholipid release resulted in the inactivation of daptomycin, the activity of the antibiotic in the culture supernatants was measured using a previously described zone of inhibition assay [16] (Fig. 1q–x). Daptomycin was inactivated to varying degrees by the bacteria, depending on the concentration of the antibiotic used. However, both staphylococcal strains, both enterococcal strains, *S. gordonii* and the GAS strain completely inactivated daptomycin at 5 µg ml^−1^, but GBS strains only partially inactivated the antibiotic at this concentration. At 10 µg ml^−1^ daptomycin, only the staphylococci, *S. gordonii* and the enterococci showed significant inactivation of the antibiotic, while at a concentration of 20 µg ml^−1^ daptomycin, only staphylococci and enterococci inactivated the antibiotic to any significant degree, with a loss of 30–60 % of antibiotic activity. However, despite triggering phospholipid release, at 40 µg ml^−1^ daptomycin there was relatively little (<20 %) inactivation of the antibiotic by any of the bacteria tested. Therefore, phospholipid release is finite and can be overcome with a sufficiently high dose of daptomycin.

The predominant phospholipid in the membrane of Gram-positive bacteria is phosphatidylglycerol, with much smaller quantities of cardiolipin and/or lysyl-phosphatidylglycerol also present [31]. Our previous work using purified phospholipids revealed that, at physiologically-relevant concentrations, phosphatidylglycerol is the only component of the membrane that can inactivate daptomycin [16]. Therefore, whilst we did not identify the particular species of phospholipid released from enterococci or streptococci, the ability of released lipids to inactivate daptomycin demonstrates the presence of phophatidylglycerol.

These data extend our previous finding that *S. aureus* releases phosphatidylglycerol in response to daptomycin, and that this results in inactivation of the antibiotic by revealing a very similar phenotype for *S. epidermidis*. Further, these findings also support the previous observation that *E. faecalis* releases phospholipids in response to daptomycin [18], and show that this phospholipid release correlates with daptomycin inactivation and bacterial survival. Streptococci, particularly *S. gordonii*, also released phospholipids and inactivated daptomycin, albeit less efficiently than *E. faecalis*. Therefore, daptomycin-induced phospholipid release appears to be a conserved mechanism across Gram-positive pathogens.

Next, we wanted to explore whether the mechanism of phospholipid release and daptomycin inactivation by enterococci and streptococci was similar to that of *S. aureus*. Therefore, we undertook further experiments with *E. faecalis,* which was the most efficient of the enterococci and streptococci at releasing phospholipids and inactivating daptomycin, and *S. aureus,* in which daptomycin-triggered phospholipid release has been well characterized [16].

In *S. aureus*, daptomycin-triggered phospholipid release is an active process that requires energy, as well as protein and lipid biosynthesis [16]. To determine whether phospholipid release by *E. faecalis* exposed to daptomycin was occurring via an active process, or simply as a consequence of membrane damage caused by the antibiotic, bacteria were exposed to the antibiotic in the presence or absence of a sub-inhibitory concentration of the phospholipid biosynthesis inhibitor, platensimycin [17]. As described previously, the exposure of *S. aureus* to daptomycin (10 µg ml^−1^) resulted in increased phospholipid in the supernatant, but this was significantly reduced in the presence of platensimycin at half the MIC (0.25 µg ml^−1^) (Fig. 2a). Similarly, phospholipid was released upon the exposure of *E. faecalis* to daptomycin (10 µg ml^−1^), but this was blocked when platensimycin was present at half the MIC (0.5 µg ml^−1^) (Fig. 2b). The presence of platensimycin prevented *S. aureus* from inactivating daptomycin (Fig. 2c) and significantly reduced the ability of *E. faecalis* to inactivate daptomycin (Fig. 2d). This confirmed that daptomycin-induced phospholipid release by *E. faecalis* is an active process that requires *de novo* lipid biosynthesis and is not simply a consequence of membrane damage caused by the antibiotic. The ability of platensimycin to block phospholipid release and prevent daptomycin inactivation by *E. faecalis* also provided strong evidence that, as for *S. aureus*, daptomycin activity is blocked by the phospholipid in the supernatant. However, it was necessary to rule out an alternative hypothesis; that the loss of daptomycin activity was simply due to binding of the antibiotic to the bacterial surface.

**Fig. 2.**
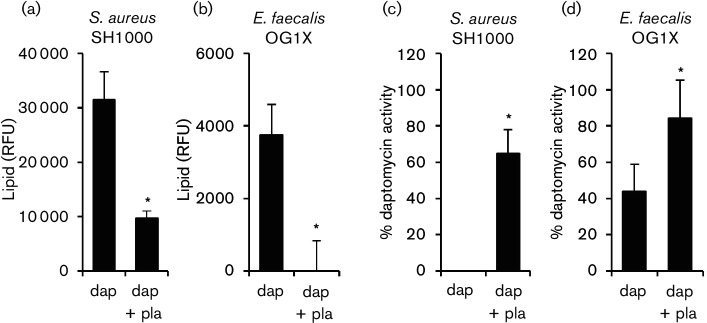
*De novo* lipid biosynthesis is required for enterococcal inactivation of daptomycin. Phospholipid concentration (RFU) in culture supernatants from *S. aureus* (a) or *E. faecalis* OG1X (b) incubated for 8 h in media containing daptomycin (10 µg ml^−1^) only (dap) or both daptomycin and 0.5 X MIC platensimycin (dap + pla). (c, d) Relative % daptomycin activity in supernatants from cultures described in (a) and (b), respectively. The data in (a) and (b) were analysed using a one-way ANOVA with Tukey’s post hoc test. The data in (c) and (d) were analysed by Student’s *t*-test. *P=<0.05.

To measure the binding of daptomycin to bacteria, daptomycin was labelled with the BODIPY fluorophore (Life Technologies) as described previously [11, 16]. As reported previously, a killing assay with *E. facealis* indicated that the labelled antibiotic had slightly reduced bactericidal activity relative to unlabelled daptomycin [11] (Fig. 3a). However, as described above for unlabelled antibiotic (Fig. 1q, x), the activity of the antibiotic decreased after incubation with *E. faecalis* or *S. aureus* (Fig. 3b), confirming that the BODIPY label does not significantly affect the interaction of the antibiotic with the bacteria studied.

**Fig. 3.**
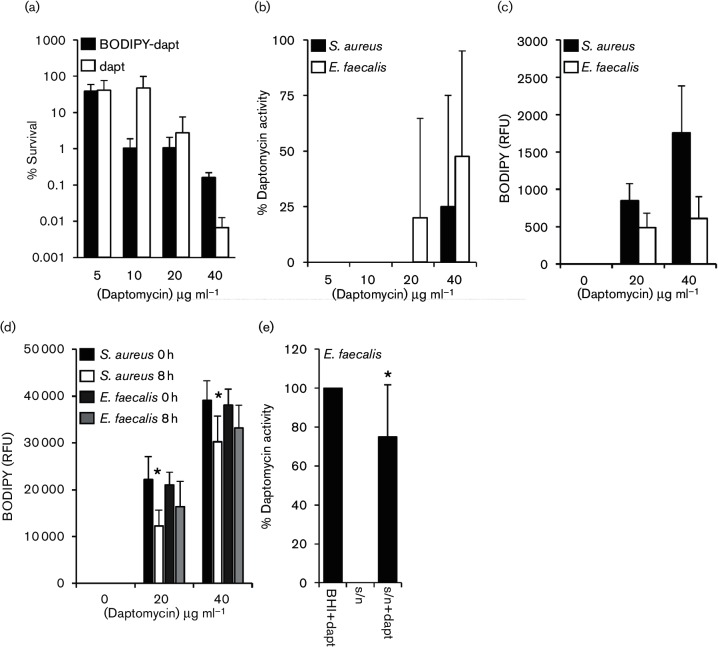
Loss of daptomycin activity in supernatant is not due to antibiotic binding to bacteria. (a) Percentage survival of *E. faecalis* OG1X incubated with various concentrations of daptomycin (dapt) or BODIPY–daptomycin (BODIPY–dapt) for 8 h. (b) Relative percentage daptomycin activity in culture supernatants described in (a). (c) Binding of Bodipy-daptomycin to *S. aureus* or *E. faecalis* OG1X after 8 h incubation in media containing the indicated concentration of the labelled antibiotic. (d) Quantification of BODIPY–daptomycin (RFU) remaining in culture supernatants from *S. aureus* or *E. faecalis* OG1X after 8 h incubation with BODIPY–daptomycin as described in (c). * indicates significantly different from 0 h time point. (e) Relative percentage activity of daptomycin (5 µg ml^−1^) activity in BHI only (BHI+dapt), in the supernatant from *E. faecalis* incubated with daptomycin for 8 h (s/n) and after the addition of 5 µg ml^−1^ daptomycin to the supernatant from *E. faecalis* incubated with daptomycin for 8 h (s/*n*+dapt). The data in (d) and (e) were analysed by a two-way ANOVA with Tukey’s post hoc test. The graphs show the mean average and, where shown, error bars represent the sd of the mean. For each panel *P=<0.05.

After 8 h incubation with BODIPY–daptomycin, the bacterial cells were pelleted and the fluorescence of both the cells and the supernatants was measured separately using a Tecan microplate reader with excitation at 502 nm and emission at 510 nm. Antibiotic attachment to the *E. faecalis* cellular fraction was similar for both BODIPY–daptomycin concentrations examined, suggesting saturated binding to cells (Fig. 3c). However, most of the antibiotic remained in the supernatant (Fig. 3d). By comparison, BODIPY–daptomycin bound *S. aureus* more strongly than *E. faecalis*, with higher levels of fluorescence associated with bacterial cells and a corresponding drop in the fluorescence of the supernatant (Fig. 3c, d). This difference in antibiotic binding may explain why the daptomycin MIC of the *E. faecalis* strains used here (2–4 µg ml^−1^) is higher than that of the *S. aureus* strain examined (1 µg ml^−1^), and why daptomycin triggers greater phospholipid release from staphylococci than enterococci.

Together, these data confirmed that the loss of daptomycin activity in *E. faecalis* cultures was not due to binding of the antibiotic to the bacterial surface or the plastic vessels used in the assays. However, as a final confirmation that phospholipids released from *E. faecalis* inactivated daptomycin, we exposed the bacterium to daptomycin (5 µg ml^−1^) to trigger phospholipid release, collected the cell-free culture supernatant and added a second dose of the antibiotic (5 µg ml^−1^). The culture supernatant containing the released phospholipids significantly reduced the activity of the second dose of daptomycin (by ~25 %; Fig. 3e). Therefore, as described for *S. aureus*, the release of phospholipids by *E. faecalis* in response to daptomycin inactivates the antibiotic. The data described above also indicate that several species of streptococci release phospholipids in response to daptomycin, and these inactivate the antibiotic, albeit to a lesser extent than for *E. faecalis* or *S. aureus.*

Streptococci and enterococci cause a range of serious diseases, including septicaemia and endocarditis, which can be treated by daptomycin, especially when the pathogen is multi-drug resistant (e.g. VRE or VISA) or the patient has a β-lactam allergy [1, 32]. Our finding that this defence mechanism is present in a variety of clinically relevant Gram-positive bacteria indicates that it is conserved and could be a viable target to improve the effectiveness of daptomycin therapy against these pathogens. However, it should be noted that this study employed drug-sensitive strains and so further work is required to explore how the phospholipid release system works in bacteria that are resistant to vancomycin or daptomycin, which typically results in alterations to the cell membrane or wall [1, 2]. Changes to the membrane may also occur *in vivo* due to the utilization of host-derived fatty acids or in response to environmental stress, such as the presence of antimicrobial peptides, and so these factors will also need to be considered [31, 33, 34].

In this work, we focussed on daptomycin because it is a last-resort antibiotic and is associated with high rates of treatment failure. However, whilst daptomycin use is increasing, it is very unlikely to have provided the selection pressure for the evolution of the phospholipid release defence mechanism described here and previously [16]. Since cationic antimicrobial peptides (CAMPs) act via a similar mechanism to daptomycin in targeting the Gram-positive cell membrane [5] we hypothesize that these host defence molecules have likely driven the evolution of phospholipid release as a defence mechanism.

The discovery of phospholipid release in several Gram-positive pathogens has expanded our growing appreciation of the broad-spectrum extracellular defence mechanisms that protect bacteria against antibiotics or host defences. For example, previous work has shown that the production of outer-membrane vesicles by *Escherichia coli* can protect against membrane-acting antimicrobials such as polymixin E and colistin [35], whilst another report revealed that lipochalins released by *Burkholderia* can sequester several different antibiotics [36]. These findings underline the complex nature of innate antibiotic resistance, but also provide opportunities for mechanistic insights and improved therapeutic approaches. For example, in this report and previously, we have shown that inhibition of phospholipid biosynthesis using platensimycin prevents the inactivation of daptomycin by both *S. aureus* and *E. faecalis* [16]. Although platensimycin has not entered clinical trials due to poor pharmacokinetic properties [17, 37], other inhibitors of lipid biosynthesis are in clinical development [38]. Therefore, the use of daptomycin in combination with lipid biosynthesis inhibitors may provide an effective way of enhancing treatment outcomes compared to the lipopeptide antibiotic alone.

In summary, we have demonstrated that *E. faecalis* releases phospholipids in response to daptomycin via an active mechanism requiring *de novo* lipid biosynthesis and that these phospholipids inactivate daptomycin. Pathogenic streptococci also appear to be capable of inactivating daptomycin by releasing phospholipids, indicating that this mechanism is conserved amongst Gram-positive pathogens.

## References

[1] Humphries RM, Pollett S, Sakoulas G (2013). A current perspective on daptomycin for the clinical microbiologist. Clin Microbiol Rev.

[2] Purrello SM, Garau J, Giamarellos E, Mazzei T, Pea F (2016). Methicillin-resistant *Staphylococcus aureus* infections: a review of the currently available treatment options. J Glob Antimicrob Resist.

[3] Seaton RA, Gonzalez-Ruiz A, Cleveland KO, Couch KA, Pathan R (2016). Real-world daptomycin use across wide geographical regions: results from a pooled analysis of CORE and EU-CORE. Ann Clin Microbiol Antimicrob.

[4] Public Health England (2016). *English Surveillance Programme for Antimicrobial Utilisation and Resistance (ESPAUR*).

[5] Straus SK, Hancock RE (2006). Mode of action of the new antibiotic for gram-positive pathogens daptomycin: comparison with cationic antimicrobial peptides and lipopeptides. Biochim Biophys Acta.

[6] Muraih JK, Pearson A, Silverman J, Palmer M (2011). Oligomerization of daptomycin on membranes. Biochim Biophys Acta.

[7] Muraih JK, Harris J, Taylor SD, Palmer M (2012). Characterization of daptomycin oligomerization with perylene excimer fluorescence: stoichiometric binding of phosphatidylglycerol triggers oligomer formation. Biochim Biophys Acta.

[8] Taylor SD, Palmer M (2016). The action mechanism of daptomycin. Bioorg Med Chem.

[9] Silverman JA, Perlmutter NG, Shapiro HM (2003). Correlation of daptomycin bactericidal activity and membrane depolarization in *Staphylococcus aureus*. Antimicrob Agents Chemother.

[10] Cotroneo N, Harris R, Perlmutter N, Beveridge T, Silverman JA (2008). Daptomycin exerts bactericidal activity without lysis of *Staphylococcus aureus*. Antimicrob Agents Chemother.

[11] Pogliano J, Pogliano N, Silverman JA (2012). Daptomycin-mediated reorganization of membrane architecture causes mislocalization of essential cell division proteins. J Bacteriol.

[12] Müller A, Wenzel M, Strahl H, Grein F, Saaki TN (2016). Daptomycin inhibits cell envelope synthesis by interfering with fluid membrane microdomains. Proc Natl Acad Sci USA.

[13] Pader V, Edwards AM (2017). Daptomycin: new insights into an antibiotic of last resort. Future Microbiol.

[14] Seaton RA, Menichetti F, Dalekos G, Beiras-Fernandez A, Nacinovich F (2015). Evaluation of effectiveness and safety of high-dose daptomycin: results from patients included in the European Cubicin® outcomes registry and experience. Adv Ther.

[15] Tran TT, Munita JM, Arias CA (2015). Mechanisms of drug resistance: daptomycin resistance. Ann N Y Acad Sci.

[16] Pader V, Hakim S, Painter KL, Wigneshweraraj S, Clarke TB (2016). *Staphylococcus aureus* inactivates daptomycin by releasing membrane phospholipids. Nat Microbiol.

[17] Wang J, Soisson SM, Young K, Shoop W, Kodali S (2006). Platensimycin is a selective FabF inhibitor with potent antibiotic properties. Nature.

[18] Wale LJ, Shelton AP, Greenwood D (1989). Scanning electron microscopy of *Staphylococcus aureus* and *Enterococcus faecalis* exposed to daptomycin. J Med Microbiol.

[19] Biagini M, Garibaldi M, Aprea S, Pezzicoli A, Doro F (2015). The human pathogen *Streptococcus pyogenes* releases lipoproteins as lipoprotein-rich membrane vesicles. Mol Cell Proteomics.

[20] Surve MV, Anil A, Kamath KG, Bhutda S, Sthanam LK (2016). Membrane vesicles of group B *Streptococcus* disrupt feto-maternal barrier leading to preterm birth. PLoS Pathog.

[21] Uhlmann J, Rohde M, Siemens N, Kreikemeyer B, Bergman P (2016). LL-37 triggers formation of *Streptococcus pyogenes* extracellular vesicle-like structures with immune stimulatory properties. J Innate Immun.

[22] Horsburgh MJ, Aish JL, White IJ, Shaw L, Lithgow JK (2002). SigmaB modulates virulence determinant expression and stress resistance: characterization of a functional *rsbU* strain derived from *Staphylococcus aureus* 8325-4. J Bacteriol.

[23] Zhang YQ, Ren SX, Li HL, Wang YX, Fu G (2003). Genome-based analysis of virulence genes in a non-biofilm-forming *Staphylococcus epidermidis* strain (ATCC 12228). Mol Microbiol.

[24] Molinari G, Talay SR, Valentin-Weigand P, Rohde M, Chhatwal GS (1997). The fibronectin-binding protein of *Streptococcus pyogenes*, SfbI, is involved in the internalization of group A streptococci by epithelial cells. Infect Immun.

[25] Wessels MR, Paoletti LC, Rodewald AK, Michon F, Difabio J (1993). Stimulation of protective antibodies against type Ia and Ib group B streptococci by a type Ia polysaccharide-tetanus toxoid conjugate vaccine. Infect Immun.

[26] Wilson CB, Weaver WM (1985). Comparative susceptibility of group B streptococci and *Staphylococcus aureus* to killing by oxygen metabolites. J Infect Dis.

[27] Cisar JO, Kolenbrander PE, Mcintire FC (1979). Specificity of coaggregation reactions between human oral streptococci and strains of *Actinomyces viscosus* or *Actinomyces naeslundii*. Infect Immun.

[28] Jacob AE, Hobbs SJ (1974). Conjugal transfer of plasmid-borne multiple antibiotic resistance in *Streptococcus faecalis* var. *zymogenes*. J Bacteriol.

[29] Ike Y, Craig RA, White BA, Yagi Y, Clewell DB (1983). Modification of *Streptococcus faecalis* sex pheromones after acquisition of plasmid DNA. Proc Natl Acad Sci USA.

[30] Clinical and Laboratory Standards Institute (2012). Methods for Dilution Antimicrobial Susceptibility Tests for Bacteria That Grow Aerobically;.

[31] Sohlenkamp C, Geiger O (2016). Bacterial membrane lipids: diversity in structures and pathways. FEMS Microbiol Rev.

[32] King A, Phillips I (2001). The *in vitro* activity of daptomycin against 514 gram-positive aerobic clinical isolates. J Antimicrob Chemother.

[33] Harp JR, Saito HE, Bourdon AK, Reyes J, Arias CA (2016). Exogenous fatty acids protect *Enterococcus faecalis* from daptomycin-induced membrane stress independently of the response regulator LiaR. Appl Environ Microbiol.

[34] Sen S, Sirobhushanam S, Johnson SR, Song Y, Tefft R (2016). Growth-environment dependent modulation of *Staphylococcus aureus* branched-chain to straight-chain fatty acid ratio and incorporation of unsaturated fatty acids. PLoS One.

[35] Manning AJ, Kuehn MJ (2011). Contribution of bacterial outer membrane vesicles to innate bacterial defense. BMC Microbiol.

[36] El-Halfawy OM, Klett J, Ingram RJ, Loutet SA, Murphy ME (2017). Antibiotic capture by bacterial lipocalins uncovers an extracellular mechanism of intrinsic antibiotic resistance. MBio.

[37] Martens E, Demain AL (2011). Platensimycin and platencin: promising antibiotics for future application in human medicine. J Antibiot.

[38] Yao J, Rock CO (2016). Bacterial fatty acid metabolism in modern antibiotic discovery. Biochim Biophys Acta.

